# Can We Unambiguously Define the Dipole Moment of Molecules in the Condensed Phase?

**DOI:** 10.3390/molecules30071539

**Published:** 2025-03-30

**Authors:** Imre Bakó, Szilvia Pothoczki

**Affiliations:** 1 HUN-REN Research Centre for Natural Sciences, Magyar Tudósok Körútja 2, H-1117 Budapest, Hungary; 2 HUN-REN Wigner Research Centre for Physics, Konkoly Thege M. út 29-33, H-1121 Budapest, Hungary; pothoczki.szilvia@wigner.hun-ren.hu

**Keywords:** dipole moment, condensed phase, PCM, localized orbital

## Abstract

Various theoretical methods were applied and evaluated to determine the dipole moment of polar protic (methanol, ethanol) and aprotic (acetonitrile, pyridine, acetone) dipoles in the crystal phase. In mono-alcohols, the dipole moment is influenced by the hydrogen bonding (H-bonding) environment, similarly to earlier findings with liquid water. Using localization techniques without considering the effect of neighboring molecules gives similar results for the dipole moment of mono-alcohols than those obtained from the polarized continuum model (PCM). However, the PCM for polar aprotic molecules provides significantly different dipole moment values compared to localization methods. Our results clearly show that the magnitude of the dipole moment in the condensed phase cannot be unambiguously determined.

## 1. Introduction

In a polar molecule, one part of the molecule is positively charged, while another part is negatively charged, leading to a molecule having an electric dipole moment. This measurable quantity, the first spatial momentum of the charge distribution, is often used to describe the accuracy of various quantum chemical methods. Many review articles indicate that the widely used DFT functionals are suitable for determining the dipole moment, as the calculated values differ by only 3–5% from experimental results or high-level quantum chemical calculations (like CCSD or CCSD (T)) [1,2,3,4,5,6]. These works recommend using the aug-cc-pVXZ(T, Q) basis set; however, the cc-pvTZ basis set is also acceptable, with a modest error of less than 5% [1,4]. Previous studies indicate that the dipole moment can be significantly affected by interactions with the molecule’s surrounding environment [7,8,9,10,11,12,13,14,15,16,17,18,19,20,21,22,23,24,25,26,27,28,29,30,31,32,33,34,35,36,37], which may arise from electrostatic charge interactions and various quantum chemical interactions, including hydrogen bonding [22,23,24,25,29,30].

Three different approaches exist in the literature to describe the changes in dipole moment caused by the aforementioned interactions. The simplest treatment of medium interaction is the polarizable continuum model (PCM), in which a continuous medium through a fixed dielectric constant interacts with a molecule [31,32,33]. The interaction with a quantum chemically treated central molecule is described using the Poisson–Boltzmann equation or the generalized Born approach. The result of these efforts is that nowadays, PCM, with all its different variants [31,32,33,34,35,36,37], is the default choice in many computational codes to couple a quantum–mechanical (QM) description of a molecular system with a continuum description of the environment.

In the second case, the central molecule is handled by a quantum chemical method of appropriate precision, while other molecules in the vicinity are treated by point charges, dipoles, or multipoles. This is a QM/MM method [8,9,10,11,12,13,16,17]. The position of the molecules acting on the surroundings relative to the central molecule can be determined from simulations (Monte Carlo or Molecular Dynamics simulation). In these cases, selecting the appropriate configurations from the simulations is important.

Thirdly, the electron density can be determined on the given molecule or smaller cluster. According to one implementation, the Bader and Voronoi [26] localization method, the 3D charge density data are used during localization, and with their help, the space to be examined is divided into disjoint parts. It has been shown earlier [26] that the sum of the charge in these disjoint volumes around the molecular center is not always 0, which also means that in the case of the calculated dipole moment (electron density first moment) the location of the center must always be given. Alternative implementation also exists by exploiting the fact that canonical (delocalized) molecular orbitals are invariant to a unitary transformation. Therefore, it is possible to apply different types of localization techniques. Several localization methods in the Hilbert space have been developed to obtain more chemically meaningful interpretations of different properties related to electron density [22,23,24,25,29,30]. Among the various processes, the most commonly used is the Boys localization [38], also known as Wannier localization in a periodic system, which is based on minimizing the spatial extent of the orbital [39]. The Pipek–Mezey method relates to maximizing Mulliken charges [40,41]. In this work, we utilize the Magnasco–Perico (MP) localization scheme [42,43,44,45], as we applied earlier [22,23,24,25], in which the Mulliken net population is maximized for an individual monomer. Several review articles discuss the applicability of these localization methods [46,47].

A typical example of the above-mentioned phenomenon was observed in the liquid state of water, where the dipole moment of water molecules significantly increases due to hydrogen bonding interactions with neighboring molecules [23,24,29,30]. An even more pronounced effect is observed around small, highly charged cations in a water environment [25]. Applying the QM/MM method, a ca. 30–50% increase was observed in the dipole moment of a water molecule in the liquid phase or water clusters compared to gas phase QM calculation (2.5–2.7 Debye (D)) [13,18,19,20]. Using the Bader method with the BLYP functional, which is widely applied in liquid simulations, the dipole moment is found to be between 2.4 and 2.45 D [22,29]. In contrast, Wannier localization (Boys) indicates this value to be around 3.0 D. Our previous studies [23,24] showed that this difference between dipole values calculated by the Bader and the localization method is independent of the used functional. Moreover, it turned out that this dipole moment depends on the number of molecules that bond to the central molecule through hydrogen bonding. We demonstrated that the dipole moment calculated for the central water molecule, using a quantum chemistry approach that considers the entire system, aligns closely with the dipole moment obtained by treating only the first sphere exactly. In this latter approach, the influence of more distant molecules is incorporated through the polarizable medium method [22]. At least for water molecules in medium-sized clusters, the change in dipole moment caused by a change in geometry is negligible [22].

Experimental works [28,48,49] also explore an increment of the dipole moment of water due to the interaction between molecules with dipole moment and their environments in its condensed phases (or clusters of several molecules). The experimental values are ca. 3.0 D and 2.66 D in liquid state and crystal phase, respectively [28,48,49]. These values contain significant uncertainties because of the approximations that were used [49]. In contrast, the dipole moment in the gas phase (1.8546 D) is well established [50].

However, certain solvents, such as simple alcohols, which are noteworthy due to their polarity, were not extensively studied in this regard. The dipole moment of methanol molecules in the liquid phase is the most accurately explored by ab initio molecular dynamics simulations [51,52,53,54,55,56,57,58,59,60]. The results obtained using the Wannier localization method are in the range of 2.53–2.84 D [53,57,60]. A deeper examination of the published data suggests that the dipole moment is significantly higher when the number of methanol molecules is less than 50 or when the density is considerably higher than the experimental value. Recent results give a dipole moment value between 2.53 and 2.60 D [57]. They found that the dipole moment depends on the number of H-bonded neighbors as in the case of AIMD simulations of liquid water.

Early QM/MM calculations (alcohol in alcohol) using AM1 or Hartree-Fock (HF) level of quantum chemistry give a value of 2.06–2.36 D [60,61,62]. More accurate calculations using the SCEE (Self-Consistent Electrostatic Embedding) method yield a value around 2.6 D [16,17]. Studies by Jorge et al. [16] for mono-alcohols with n = 10 long carbon atoms give a difference between liquid and gas phase dipole moment of 0.90–0.92 D. These methods do not take into account quantum chemical effects due to H-bonding (e.g., electron transfer to the nonbonding orbital). Here, we would like to remark that the dipole moments of liquid methanol from AIMD calculation and QM/MM methods are similar, probably thanks to the error cancelation.

In the early development of non-polarizable force fields, gas-phase calculations at the HF/6-31G* level were used with the assumption that this method significantly overestimates the dipole moment of the alcohol molecule (about 10%) [17,60,61,62]. The dipole moment for methanol resulted between 1.94 and 2.14 D [61,62,63]. If a polarizable model was used the dipole moment increased by about 0.7 D [63,64,65]. Later on, the charge distributions were calculated using polarizable continuum models (PCM) [31,32,33,34,35,36,37], in which the dielectric constant of the liquid phase was applied. In several cases based on the so-called bisector method, the charge distributions were obtained taking into account both gas and liquid phase calculations [17].

Another important group of solvents includes acetonitrile (methyl cyanide), pyridine, and acetone, which are aprotic organic molecules with a significant permanent dipole moment. The primary factor in determining the structure of the condensed phase is the intense competition between dispersion, electrostatic, and polarization interactions [66,67,68,69,70,71]. Numerous simulation studies indicate that considering polarization interactions in the liquid phase is essential [11,70,71,72,73,74,75,76,77,78,79]. As a result, an increase in dipole moment was observed approximately 0.5 to 1.5 D for acetonitrile [11,70,71,72,73,74], and around 1.0 D [75,76,77,78,79,80,81] for both pyridine and acetone. On the other hand, the different types of H-bonding interactions (CH…N, CH…O, and CH…π) are also crucial. In the case of pyridine, it is important to note that the nuclear quantum effect also plays an important role in determining the crystal structure. 

The experimental results from far-infrared optical constant measurements imparted 4.5 D in the liquid phase of acetonitrile [82]. In the case of liquid acetone, the dipole moment fluctuates around 3.3 D with a half-width of about 0.5 D [9].

In this paper, we explore how the dipole moment varies in molecular clusters composed of different protic (methanol, ethanol) and aprotic (acetonitrile, pyridine, acetone) molecules using three DFT functionals (M05-2X, M06-2X, and ωB97XD) to calculate the dipole moment through Magnasco–Perico (MP) localization. The molecular clusters are derived from the crystal structures of the molecules. Our additional aim is to investigate the effect of the polar continuum approximation on the dipole moment of certain protic molecules. Finally, we pay particular attention to describing how the dipole moment depends on the number of H-bonded neighbors in the protic H-bonded systems.

## 2. Results and Discussion

### 2.1. Mono-Alcohols

Mono-alcohols are simple organic molecules that contain a hydrophilic OH group, which has two H-bond acceptors and one H-bond donor capability, along with a hydrophobic group. In this study, we focus on the two simplest members of this group namely: methanol and ethanol. These systems show a strong preference for forming endless H-bond chains [83].

The values of dipole moment for one molecule in the gas phase were calculated by three different DFT functionals (M05-2X [84], M06-2X [85], and ωB97XD [86]) using the cc-pVTZ and AUG-cc-pVTZ basis sets. Furthermore, we applied the most advanced (highest precision) CCSD/AUG-cc-pVTZ and CCSD(T)/AUG-cc-pVTZ (AUTZ) methods to validate the chosen electronic structure approaches. As reference calculations polarizable continuum model (PCM) [35,36,37] and SMD [37] models were used. Some comprehensive review articles on these methods can be found in Refs. [31,32,33,34]. The results for methanol and ethanol molecules are in Table 1.

The calculated values for monomers are in good agreement with experimental values. The dipole moments obtained from the PCM are approximately 0.3 D and 0.55 D larger for methanol and ethanol, respectively, than those from the monomer calculation in the gas phase. In the case of the SMD model, the difference is even higher: 0.4 D for methanol and 0.6 D for ethanol. The dipole moment values using PCM and SMD methods are similar to those calculated by applying polarized force fields [60,61,62]. The PCM and SMD methods describe electrostatic interactions similarly, but they differ in how they treat the size of atoms. It is important to note that neither PCM nor SMD can properly handle H-bond interactions.

For methanol, 2, 4, 8, 10, and 12 molecules were investigated by the M05-2X/cc-pVTZ level of theory. The crystal phase was chosen as the starting point to most easily monitor the effect of the molecular environment. Monomer geometry was taken from the crystal phase. Their coordinates can be found in the Supporting Information. It is worth recalling that in methanol, only one characteristic H-bonded distance can be detected [87].

**Figure 1:**
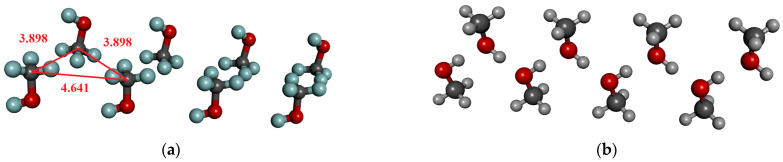
H-bonded and not H-bonded chain of methanol consisting of 8 molecules from the crystal structure. (**a**) Directional but not H-bonded molecular arrangements; (**b**) H-bonded chain.

We have to draw attention to the existence of such permanent structures in the crystal (see Figure 1a), where the dipole moment of the molecules is unidirectional, but there is no H-bonding interaction between them. However, results in Table 2 are shown only for molecular arrangements that can form hydrogen bonds (see Figure 1b). It is important to note that in a cluster of eight molecules, where there is no H-bonded interaction between the molecules, the average dipole moment (1.74 ± 0.01) increases by approximately 0.03 D compared to the dipole moment of optimized geometry.

**Figure 2:**
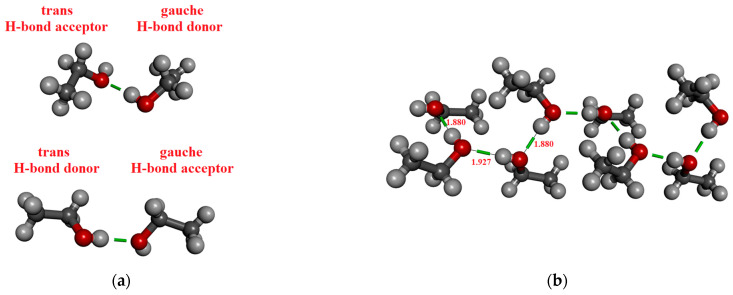
H-bonded configurations of ethanol. (**a**) Two types of optimized H-bonded dimers. (**b**) H-bonded chain structure consisting of eight molecules from the crystal structure.

In the case of ethanol, 2, 4, 8, 10, and 12 molecules were considered by the M05-2X/cc-pVTZ level of theory. Table 3 contains the results. The coordinates derived from the crystal structure can be found in the Supporting Information. It is important to highlight that two different dimers of ethanol occur in the crystal phase, depending on whether the molecule, which acts as an H-bond donor is in a trans (cf. Figure 2a lower panel) or gauche (cf. Figure 2a upper panel) conformation. Accordingly, two distinct H-bonded distances exist, corresponding to the trans-donor (1.914 Å) or gauche-donor (1.887 Å) conformers [88]. Note that the dipole moment of ethanol differs slightly between its trans and gauche conformations, 1.781 D and 1.723 D, respectively. It is about 0.1–0.15 D larger than the dipole moments of optimized ethanol monomer. This difference in dipole moments is due to the difference in geometries (bond lengths and angles).

Both for methanol and ethanol, the dipole moment of the molecules increases as the chain length increases. However, for methanol, regardless of the applied localization methods (MP or MP_trunc), it can be observed that beyond a certain length, namely the eight-member chain, the dipole moment hardly increases; instead, the value converges. In the case of ethanol, the magnitude of the dipole moment remains constant for configurations containing eight or more molecules using MP_trunc localization. With MP localization, the dipole moment converges at slightly longer chain lengths.

It was found that the dipole moment values are significantly influenced by the H-bonded state of the molecule (0A1D, 1A0D, 1A1D), as presented in Figure 2b. The highest values are observed for the 1A1D case for both methanol and ethanol. Recently, Marx et al. [57], based on AIMD simulations, had similar findings, that the dipole moment of the methanol molecules in the 1A0D and 1A1D H-bonded states is significantly higher than those in the 0A1D state. In the case of methanol, our calculated values agree well with the size of the dipoles calculated by the Wannier localization method for liquid AIMD simulations containing large (N > 64) methanol molecules [57,60].

For ethanol, it can be observed that without taking into account the long tail term of the orbitals from other monomers (MP_trunc), the calculated dipole moments are significantly lower than in the MP models. A similar phenomenon, namely that the two types of localization techniques give different results, has already been observed in water clusters [22,23] and liquid water [23]. The largest difference occurs for molecules, which are in the middle of the chain (1A1D). Additionally, this H-bonded state (1A1D) has the highest dipole moment. It may be noted here that the value of dipole moments obtained using the SMD method (Table 1) agrees well with those obtained using the MP_trunc localization technique, in which the “long tail” part of localization orbitals is omitted.

**Figure 3:**
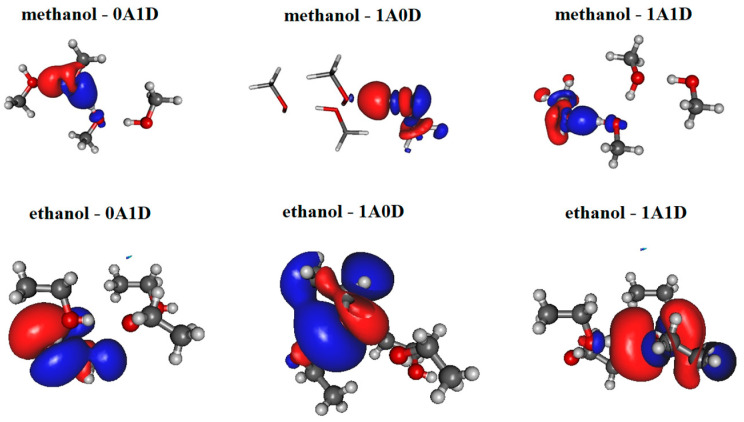
Localized molecular orbitals from methanol and ethanol clusters consisting of four molecules using Magnasco–Perico localization.

The most significant localized molecular orbitals associated with the charge transfer contribution are presented in Figure 3 for all studied H-bonded states (0A1D, 1A0D, 1A1D) of both methanol and ethanol. The magnitude of the charge transfer term from the MP localization technique is 0.06–0.07/e/for methanol, 0.035–0.04/e/for ethanol dimers with H-bond acceptor in trans configuration (cf. Figure 2a upper panel), and 0.032–0.04/e/for ethanol dimers with H-bond donor in trans configuration (cf. Figure 2a lower panel). In the case of 1A0D and 1A1D, the contribution from the adjacent donor molecule can be clearly observed (usually from H_O_ 3S orbital). This type of delocalization effect can explain why the dipole moment of molecules in these states is significantly larger.

### 2.2. Aprotic Molecule with High Dipole Moment

#### 2.2.1. Acetonitrile

For the aprotic polar molecules we have studied, the H-bonding interaction does not play a dominant role in determining the crystal structure. In these cases, electrostatic and steric interactions are the structure determinants. The calculated dipole moments in both the vacuum and the two PCM do not significantly depend on the method used or the basis set. Here, we would like to point out that hereinafter for all three studied aprotic polar molecules (acetonitrile, pyridine, and acetone) the dipole moment value calculated in vacuum at the M05-2X/cc-pVTZ level was taken as the reference value.

Using different PCM and SMD models, the dipole moment is higher by 0.9–1.1 D compared to the monomer (Table 4). The SMD method gives the highest polarization effect with 0.15–0.25 D larger dipole moments than the PCM. The dipole moment changes due to the difference in geometries between the optimized geometry and the crystal phase 0.02 D.

**Figure 4:**
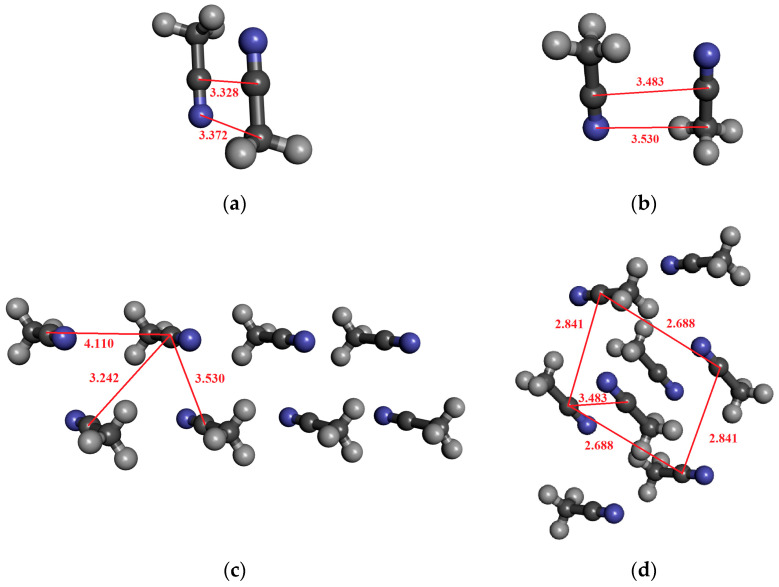
The investigated structures of acetonitrile: (**a**) optimized dimer with anti-dipole orientation; (**b**) anti-dipole orientation from crystal structure; (**c**,**d**) two characteristic acetonitrile clusters in anti-dipole arrangements from crystal structures consisting of eight molecules.

For acetonitrile, dimeric, tetrameric, and octameric structures were investigated in which the molecules are arranged in an anti-dipole arrangement. Table 5 contains the results. The coordinates derived from the crystal structure can be found in the Supporting Information. The α-form of acetonitrile crystal forms a perfectly ordered monoclinic crystal structure, in which a chain of head-to-tail ACN molecules is observed, running alongside four other antiparallel chains [89]. It is important to highlight that the C–N distance in the crystal is significantly longer (0.2–0.3 Å) than for the optimized dimer (cf. Figure 4a,b). Here, we consider both cases in the calculations.

For the optimized dimer structure (Figure 4a), the dipole moment is about 0.5 D higher, than those from the monomer in the vacuum phase. Furthermore, the difference (0.05 D) between the two localization techniques (MP and MP_trunc) indicates the existence of a weak H-bond of type CH…N.

In the case of the structure shown in Figure 4c, where the eight molecules are in antiparallel arrangements, the dipole moment is higher by about 0.36 D, and the H-bonding effect is negligible. On the other hand, for the structure shown in Figure 4d, the octamer structures, where each of the four central acetonitrile molecules interact by two CH…N H-bonds, the dipole moment difference between the MP and MP_trunc localization methods is 0.1–0.13 D. Both values, 4.67 D for MP and 4.57 D for MP_trunc, are higher than those in the gas phase, which is 4.095 D. Additionally, the MP value is greater than what we obtained for the optimized dimer (Figure 2a), where the C…N distance is significantly shorter. The fact that the dipole moment is larger in that system, where the C…N distances are larger can be attributed to the existence of the H-bonding.

**Figure 5:**
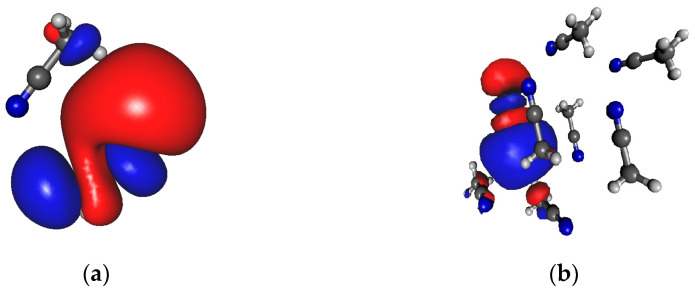
(**a**) Magnasco–Perrico localized orbital for the optimized dimer. (**b**) Magnasco–Perico localized orbital from acetonitrile structure consisting of eight molecules.

The corresponding most dominant orbital (related to the long tail part of the orbital) is shown in Figure 5. According to the results shown in Table 5, the existence of a weak H-bond of type CH…N can be observed.

The calculated values obtained from the PCM and SMD models are significantly higher than those of the MP and MP_trunc localization techniques. The values obtained from the more exact QM methods (MP and MP_trunc) agreed well with the so-called “half-charged” model [17].

#### 2.2.2. Pyridine

The results obtained for pyridine (Table 6) are quite similar to those reported for acetonitrile (Table 4). The calculated value of the dipole moment is almost the same regardless of the electronic structure method we used. The PCM and SMD models yield higher values than those obtained from monomer calculations. The dipole moment changes due to the difference in geometries between the optimized geometry and the crystal phase 0.04 D.

**Figure 6:**
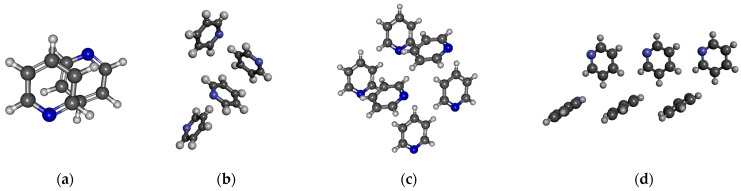
Investigated structures of pyridine: (**a**) anti-dipole orientation of optimized dimer pyridine structure; (**b**) pyridine cluster consisting of four molecules; (**c**,**d**) two different arrangements of pyridine cluster consisting of six molecules.

For pyridine, four different structures were studied (Figure 6). One is the well-known anti-dipole structure of dimer with a plane–plane distance of around 3.3 Å (Figure 6a). In the crystal phase, such a structure does not exist. The other three structures were selected from the crystal. In the structures comprising four and six pyridine molecules, as depicted in Figs. 6b and 6c, both L-type and T-type configurations are observed. These configurations indicate the presence of quadrupole–quadrupole interactions. Additionally, CH…N and CH…π interactions can also be identified. In Figure 6d, besides the L structure typical of a quadrupole–quadrupole interaction, only the presence of the CH…N H-bond can be recognized. The calculated average dipole moments, together with their standard deviation, are shown in Table 7.

**Figure 7:**
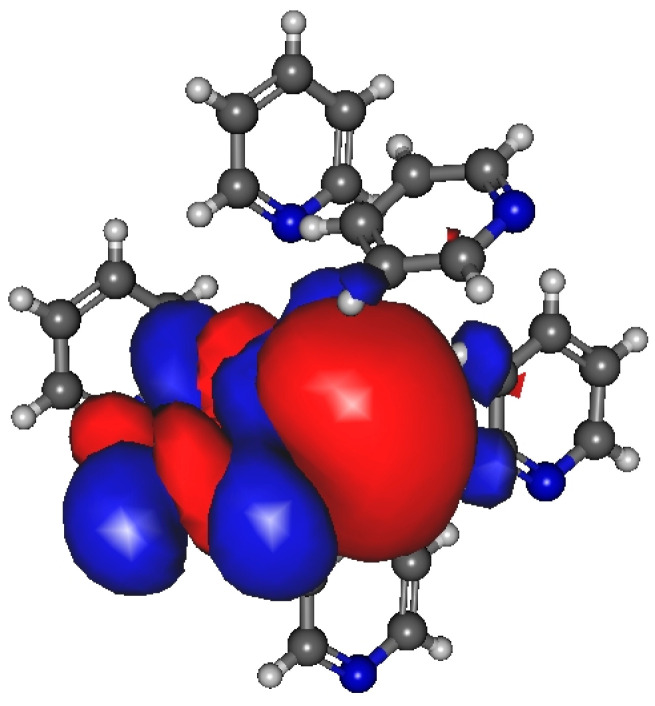
Localized molecular orbital using Magnasco–Perico localization from pyridine molecular cluster consists of 6 molecules.

For the minimum energy dimer, no significant amount of charge transfer can be found (0.02/e/). However, Figure 7 shows an increase in electron density associated with the “cage” critical point on the density difference curves for the structure shown in Figure 6c. In the case of the structure shown in Figure 6b–d, the existence of the H bond is confirmed by the difference in dipole moments obtained by MP and MP_trunc. The average dipole moment increase is 0.2–0.3 D compared to the gas phase. More detailed investigation showed that the dipole moment of a pyridine molecule when it acts as an H-bond acceptor has a 0.2–0.3 D larger dipole moment than those molecules that donate the H-bond.

For pyridine, it is also true that the application of different types of continuum models provides a significantly larger dipole moment than we obtained through other calculations (cf. Table 6 and Table 7). More accurate QM methods (MP and MP_trunc) provide dipole momentum values that correspond to the so-called “half-charged” model [17], similarly as we found in the case of acetonitrile.

#### 2.2.3. Acetone

Table 8 contains the results obtained for acetone using various electronic structure methods. Continuum models significantly overestimate the size of the dipole momentum of the monomer. Among the systems investigated, only in this case were the values from the PCM nearly equal to those from the SMD models. Except for the AUG-cc-pVTZ basis, values from the PCM were even higher than those from the SMD model. The dipole moment changes due to the difference in geometries between the optimized geometry and the crystal phase 0.08 D.

**Figure 8:**
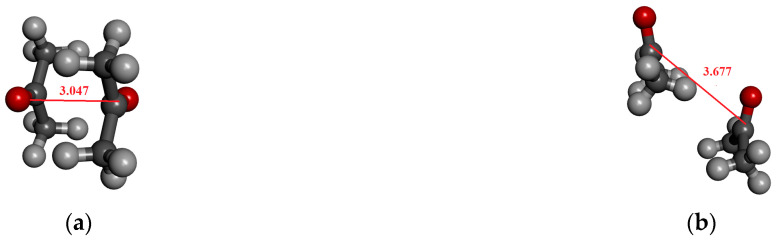

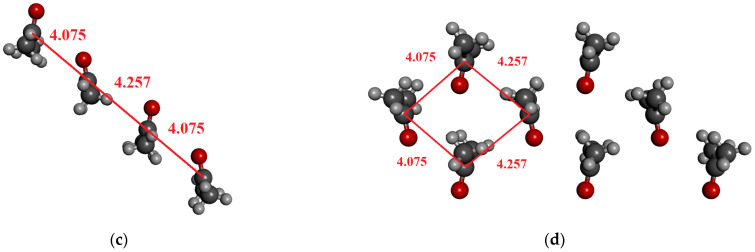
Investigated structures of acetone. (**a**) Anti-dipole orientation of optimized acetone structure; (**b**) acetone cluster consisting of two molecules from crystal; (**c**) acetone cluster consisting of four molecules; (**d**) acetone cluster consisting of eight molecules.

In the optimized dimer geometry, the C…O distance is ca. 3.05 Å while the CH…O distance is 2.65 Å. This structure corresponds to an anti-dipole dimer configuration (Figure 8a). In the not-optimized case, the C-O distance is significantly larger (Figure 8b). For the tetramer cluster (Figure 8c), originating from the crystal structure, a head-to-tail structure is obtained in which the axes of the two molecules are shifted in a parallel way. In this latter case, the characteristic C…O distances are 3.2 Å and 3.8 Å, to which the structure-defining electrostatic interaction can be assigned. Additionally, the CH…O distance for this structure is around 2.75 Å and 2.88 Å. Even for a structure of eight acetone molecules (Figure 8d), these distances can be attributed to the most important electrostatic or H-bonding interactions.

For the optimized dimer, the change in dipole moment is around 0.5 D compared to the gas phase value. The difference between the MP and MP_trunc methods in the dipole moment values for the optimized dimer is ca. 0.06 D. The increase in dipole moment can be attributed to a charge transfer of approximately 0.01/e/. The associated localized orbital can be identified, with the 3S orbital of the H_C_ serving as the donor orbital. In this case, the C…C distance is significantly shorter than the value found in the crystal structure (Table 9).

For both the tetramer and octamer structures, a difference of about 0.04–0.07 D can be seen between the dipole moment determined by the two localization methods (Table 9). This certainly suggests that CH…O interactions play a detectable role in determining the structure. Similarly to acetonitrile and pyridine, the continuum models overestimate the dipole moment obtained from MP and MP_trunc localizations.

### 2.3. Chemical Energy Component Analysis (CECA)

Additionally, we calculated the different energy components from the CECA calculation [88,90,91,92,93,94,95]. These results and the size of the charge transfer (CT) term from the MP localization are presented in Table 10. The CT term for methanol is about two times larger than in the case of ethanol.

In all investigated systems, the size of the CT term is smaller than in the case of the water cluster [22,23]. Very interestingly, the sum of the exchange and overlap interactions is about 60–70% of the electrostatic interaction. Our previous studies [22,23] have shown that this sum is about 90–95% for water clusters. The ratio of the average strength of the OH…O and CH…X terms is between 1/5th and 1/6th. This value is correlated well with the CT term obtained from the MP localization technique.

In the case of aprotic polar solvents, the size of the CT term corresponding to CH…X-type H-bonds is about 1/7th to 1/4th of that in water. The electrostatic, exchange, and overlapping terms are significantly smaller for the CH…X type of interaction than those for water.

## 3. Methods

In all cases, we used the Gaussian 09 RevE software package with the “SCF = Tight” option [96]. We performed calculations on the investigated clusters using the M05-2X [84], M06-2X [85], ωB97XD [86], CCSD, and CCSD(T) levels of theory with the cc-pVTZ and aug-cc-pVTZ basis sets. For all studied systems, the calculated dipole values using these DFT methods can be found in the Supporting Information.

The monomer and dimer geometries were optimized using the “Opt = Tight” and “int = Ultrafine” options. The vibration frequencies were calculated to prove the existence of the true minimum. For these calculations, we applied the M05-2X/cc-pVTZ level of theory. The calculated dipole moments of optimized monomers are presented in Table 11.

Our previous studies demonstrated for water clusters that the discrepancy arising from using different localization techniques to calculate a molecule’s dipole moment is minimal [22,23]. Here, we applied the Magnasco–Perico localization technique [42,43,44,45]. First, we obtain non-orthogonal localized trajectories, which are determined using the localization criterion from the Mulliken population analysis (maximum net population). The parameter used in the localization is defined by the following equation:

(1) $$M=\sum_{i}^{np}\frac{\sum_{\alpha ,\beta \in A_{i}}c_{i,\alpha }^{loc}S_{\alpha \beta }c_{i,\beta }^{loc}}{\sum_{\alpha ,\beta }c_{i,\alpha }^{loc}S_{\alpha \beta }c_{i,\beta }^{loc}}\;,$$

where *α* and *β* refer to the orbitals that belong to the atoms preassigned during the localization. The superscript “*loc*” describes the coefficient already assigned to the localization, and *S_αβ_* is the overlap matrix of *α-* and *β*-basis orbitals. The summation applies to all the preselected atoms, where np is the number of the occupied molecular orbital. The summation for *α* and *β* in the denominator is for all base orbitals, while in the numerator, it is only for the orbitals that belong to the preassigned fragment. With this localization technique, the resulting orbitals, whose number is determined by the number of electrons assigned to the localized atoms, i.e., monomers, are not orthogonal. For this to make the sum of the dipole moments calculated for the localized monomers equal to the total dipole moment, a symmetric (or Löwdin’s) orthogonalization is applied. The so-called long tail term of localized orbital (contribution from different monomers in the MP scheme) can give us information about the charge transfer term connected to the interaction among the monomers (like H-bonds) [22,23,35].

There is an additional approximation in which only the contributions belonging to the monomer are taken into account, i.e., the delocalization tail belonging to the orbitals from other monomers is completely omitted. We refer to this Magnasco–Perico truncation (MP_trunc). In our previous studies [22,23], we have shown that the dipole moments derived from this method closely align with those calculated using the Bader method, based on calculations for water clusters. It is worth pointing out, again, that in all investigated systems, the dipole moments obtained by either MP or MP_trunc show only slight dependence on the applied DFT method.

We also conducted calculations using polarizable continuum models (PCM [34,35], SMD (Solvation Model based on Density) [33]), which integrate the quantum mechanical descriptions of solutes with a continuum representation of the environment through the use of a dielectric constant. The dielectric constants used were 32.613 for methanol, 24.853 for ethanol, 35.688 for acetonitrile, 12.978 for pyridine, and 20.493 for acetone.

The Chemical Energy Component Analysis (CECA) energy decomposition method [88,90,91,92,93,94,95] was applied to analyze the interactions between monomers (CH…O type of H-bond). In this energy decomposition tool, the total energy is approximated as the sum of the atomic and diatomic energy contributions. The diatomic members can also be decomposed into electrostatics, exchange effects, diatomic overlap, and atomic basis extension contributions. In our previous work [95], we showed that the exchange and overlap contributions, which are characteristic of describing interactions between orbitals, can be very well used to detect weak H-bonds, even in cases where other methods cannot be successfully applied [93,94].

The clusters used in our calculations were derived from crystal structures determined from X-ray or neutron diffraction measurements [83,87,89,97,98,99].

## 4. Conclusions

In this work, we have demonstrated, in good agreement with previous results [57], that the dipole moment in the H-bonded structures of the mono-alcohol under investigation depends significantly on the number of H-bonded neighbors. For the MP localization technique, the dipole moment shows a significantly larger variation when the molecule participates as an acceptor than when it participates as a donor. Our calculations show that the dipole moment increase is significantly larger for methanol. Additionally, both the PCM and SMD models significantly underestimate the change in dipole moment. For ethanol, the polarizable continuum models show similar dipole moment variations to the results obtained using the localization method.

The dipole moments obtained from acetonitrile structures show that the values calculated using localization methods are approximately half of those obtained from gas-phase or polarization continuum models. In the case of pyridine, and acetone in particular, the dipole moments obtained from crystal structures (MP or MP_trunc) differ only slightly from the gas-phase (monomer) data.

In all the investigated systems, from our results described above, it is clear that the magnitude of the dipole moment of molecules in the condensed phase depends significantly on the applied localization method (MP and MP_trunc).

We show that a significant part of these changes originates from CH…X-type H-bonding. In the case of acetone, this weak H-bonding of the CH..O type is most pronounced for the optimized acetone dimer.

Our calculations indicated that the dipole geometries calculated from the optimized geometry and the crystal phase slightly differ. The magnitude of this difference is less than 0.1–0.15 D.

We have also shown, by applying the CECA energy resolution method to methanol and ethanol, that the H-bonds are of similar strengths to those observed in water clusters. The strength of the CH…X type H bonds is significantly (about 15–25%) lower than the data obtained in water clusters [21,22].

## Figures and Tables

**Table 1:** Dipole moment (D) of methanol and ethanol molecules calculated from different DFT functionals. (Experimental dipole moment of the investigated molecules: 1.701 D for methanol and 1.762 D for ethanol [25]).

Electronic Structure Methods	Systems	Monomer	PCM	SMD
M05-2X/cc-pVTZ	methanol	1.718	2.024	2.237
	ethanol	1.861	2.216	2.442
M06-2X/cc-pVTZ	methanol	1.668	1.969	2.180
	ethanol	1.808	2.156	2.379
ωB97XD/cc-pVTZ	methanol	1.634	1.932	2.142
	ethanol	1.777	2.124	2.348
M05-2X/AUG-cc-pVTZ	methanol	1.735	2.090	2.311
	ethanol	1.942	2.343	2.568
M06-2X/AUG-cc-pVTZ	methanol	1.689	2.036	2.254
	ethanol	1.891	2.283	2.504
ωB97XD/AUG-cc-pVTZ	methanol	1.658	2.011	2.231
	ethanol	1.868	2.266	2.489
CCSD/AUG-cc-pVTZ	methanol	1.678	2.033	2.245
	ethanol	1.986	2.274	2.491
CCSD(T)/AUG-cc-pVTZ	methanol	1.682	2.029	2.251
	ethanol	1.984	2.281	2.157

**Table 4:** Dipole moment (D) of the acetonitrile molecule calculated from different DFT functionals. (Experimental dipole moment of the investigated molecules: 3.92–3.95 D [25]).

Electronic Structure Methods	Monomer	PCM	SMD
M05-2X/cc-pVTZ	4.095	4.954	5.107
M06-2X/cc-pVTZ	3.998	4.837	5.142
ωB97XD/cc-pVTZ	4.020	4.871	5.344
M05-2X/AUG-cc-pVTZ	4.158	5.064	5.107
M06-2X/AUG-cc-pVTZ	3.998	4.837	5.267
ωB97XD/AUG-cc-pVTZ	3.970	4.989	5.091
CCSD/AUG-cc-pVTZ	3.987	4.796	4.991
CCSD(T)/AUG-cc-pVTZ	3.918	4.721	4.912

**Table 5:** Dipole moment (D) of acetonitrile configurations calculated by M05-2X/cc-pVTZ level of theory applying Magnasco–Perico (MP) or truncated Magnasco–Perico (MP_trunc) localizations.

Number of Molecules	MP	MP_trunc
2 (Figure 4a)	4.56	4.51
2 (Figure 4b)	3.96	3.94
4	4.40 ± 0.03	4.38 ± 0.02
8 (Figure 4c)	4.39 ± 0.02	4.36 ± 0.02
8 (Figure 4d)	4.67 ± 0.20	4.57 ± 0.21

**Table 6:** Dipole moment (D) of pyridine molecule calculated from different DFT functionals. (Experimental dipole moment of the investigated molecules: 2.27 D [25]).

Electronic Structure Methods	Monomer	PCM	SMD
M05-2X/cc-pVTZ	2.363	3.036	3.214
M06-2X/cc-pVTZ	2.290	2.943	3.117
ωB97XD/cc-pVTZ	2.322	2.992	3.169
M05-2X/AUG-cc-pVTZ	2.436	3.181	3.369
M06-2X/AUG-cc-pVTZ	2.363	3.083	3.267
ωB97XD/AUG-cc-pVTZ	2.405	3.155	3.345
CCSD/AUG-cc-pVTZ	2.423	3.175	3.368
CCSD(T)/AUG-cc-pVTZ	2.381	3.104	3.288

**Table 7:** Dipole moment (D) of pyridine configurations calculated by M05-2X/cc-pVTZ level of theory applying Magnasco–Perico (MP) or truncated Magnasco–Perico (MP_trunc) localizations.

Number of Molecules	MP	MP_trunc
2 (Figure 6a)	2.50 ± 0.01	2.49 ± 0.01
4 (Figure 6b)	2.58 ± 0.20	2.53 ± 0.20
6 (Figure 6c)	2.59 ± 0.10	2.50 ± 0.05
6 (Figure 6d)	2.60 ± 0.11	2.55 ± 0.06

**Table 8:** Dipole moment (D) of the acetone molecule calculated from different DFT functionals. (Experimental dipole moment of the investigated molecules: 2.93 D [25]).

Electronic Structure Methods	Monomer	PCM	SMD
M05-2X/cc-pVTZ	3.153	3.881	3.877
M06-2X/cc-pVTZ	3.026	3.728	3.727
ωB97XD/cc-pVTZ	3.022	3.728	3.725
M05-2X/AUG-cc-pVTZ	3.270	4.072	4.074
M06-2X/AUG-cc-pVTZ	3.145	3.920	3.926
ωB97XD/AUG-cc-pVTZ	3.146	3.932	3.937
iCCSD/AUG-cc-pVTZ	2.978	3.723	3.723
CCSD(T)/AUG-cc-pVTZ	3.068	3.827	3.833

**Table 9:** Dipole moment (D) of acetone configurations calculated by M05-2X/cc-pVTZ level of theory applying Magnasco–Perico (MP) or truncated Magnasco–Perico (MP_trunc) localizations.

Number of Molecules	MP	MP_trunc
2 (Figure 8a)	3.73	3.67
2 (Figure 8b)	3.28, 3.04	3.27, 3.04
4 (Figure 8c)	3.23 ± 0.14	3.19 ± 0.10
8 (Figure 8d)	3.19 ± 0.18	3.12 ± 0.12

**Table 10:** Different energy contributions from CECA calculations (kcal/mol) and the charge transfer term from the MP localization technique in the investigated systems. These contributions can be used as characteristic values for the strength of “classical” or CH…O H-bonds.

Systems	Electrostatic Interaction	Exchange Interaction	Overlapping Interactions	Charge Transfer Term (MP)
Methanol	−45.1	−15.7	−14.4	0.06–0.07
Ethanol (Figure 2a upper)	−30.1	−7.5	−10.5	0.035–0.04
Acetonitrile	−7.1	−2.3	−2.9	0.016
Pyridine	−6.3	−1.3	−3.1	0.02
Acetone	−5.0	−1.0	−2.8	0.01
Water cluster [22,23]	−40.1	−24.0	−20.1	0.08

**Table 2:** Dipole moment (D) of methanol configurations calculated by M05-2X/cc-pVTZ level of theory applying Magnasco–Perico (MP) or truncated Magnasco–Perico (MP_trunc) localizations.

Number of Molecules	0A1D (Chain-End) MP	0A1D (Chain-End) MP_trunc	1A0D (Chain-End) MP	1A0D (Chain-End) MP_trunc	1A1D (Inside) MP	1A1D (Inside) MP_trunc
2	1.82	1.84	2.14	1.92	−	−
4	2.02	2.02	2.38	2.05	2.48 ± 0.04	2.16 ± 0.02
8	2.05	2.05	2.41	2.08	2.60 ± 0.05	2.26 ± 0.03
10	2.06	2.05	2.42	2.08	2.63 ± 0.06	2.28 ± 0.03
12	2.06	2.06	2.42	2.08	2.65 ± 0.05	2.29 ± 0.04

**Table 3:** Dipole moment (D) of ethanol configurations calculated by M05-2X/cc-pVTZ level of theory applying Magnasco–Perico (MP) or truncated Magnasco–Perico (MP_trunc) localizations.

Number of Molecules	0A1D (Chain-End) MP	0A1D (Chain-End) MP_trunc	1A0D (Chain-End) MP	1A0D (Chain-End) MP_trunc	1A1D (Inside) MP	1A1D (Inside) MP_trunc
2 (Figure 2a upper)	1.81	1.79	2.05	1.88		
2 (Figure 2a lower)	1.79	1.76	2.04	1.82		
4	1.97	1.81	1.990	1.94	2.11 ± 0.12	2.00 ± 0.02
8	2.06	1.83	2.15	1.96	2.30 ± 0.16	2.09 ± 0.10
10	2.01	1.83	2.16	1.97	2.32 ± 0.13	2.11 ± 0.10
12	2.01	1.83	2.16	1.97	2.34 ± 0.13	2.12 ± 0.10

**Table 11:** The dipole moment (D) of optimized molecule, calculated by M05-2X/cc-pVTZ level of theory.

Systems	Dipole Moment
Methanol	1.719
Ethanol (Figure 2a upper)	1.625
Ethanol (Figure 2a lower)	1.723
Acetonitrile	4.073
Acetone	3.118
Pyridine	2.256

## Data Availability

The data presented in this study are available on request from the corresponding author.

## Supplementary Materials

The following supporting information can be downloaded at https://www.mdpi.com/article/10.3390/molecules30071539/s1. Coordinates of methanol cluster with 2, 4, 6, 8, 10, and 12 molecules; coordinates of ethanol cluster with 2, 4, 6, 8, 10, and 12 molecules; coordinated of acetonitrile cluster with 2, 4, and 8 molecules; coordinates of pyridine cluster with 2, 4, and 6 molecules; coordinates of acetone cluster with 2, 4 and 8 molecules. Dipole moment (D) of molecules calculated using different DFT methods for methanol, ethanol, acetonitrile, pyridine, and acetone.
