# Supplementary material for: Can We Unambiguously Define the Dipole Moment of Molecules in the Condensed Phase?
Source: Molecules. 2025 Mar 30;30(7):1539. doi: 10.3390/molecules30071539 (PMC11990377; doi:10.3390/molecules30071539)
Supplement: Supplementary file 1 [file molecules-30-01539-s001.zip › molecules-3513501-supplementary.pdf]

SUPPORTING INFORMATION

for

Article

Can We Unambiguously Define the Dipole Moment of Molecules in the Condensed Phase?

Imre Bakó [1,*] and Szilvia Pothoczki [2]

[1] HUN-REN Research Centre for Natural Sciences, Magyar Tudósok Körútja 2, H-1117 Budapest, Hungary
[2] HUN-REN Wigner Research Centre for Physics, Konkoly Thege M. út 29-33, H-1121 Budapest, Hungary; pothoczki.szilvia@wigner.hun-ren.hu
* Correspondence: bako.imre@ttk.hun-ren.hu

Coordinates of Methanol clusters

Methanol – cluster with 4 molecules

12

C 3.3364 13.8890 16.7987

O 4.6048 14.0393 16.2082

H 4.8436 13.2118 15.6806

H 3.1693 14.7930 17.3822

H 2.5772 13.8226 16.0654

H 3.3315 13.0749 17.4904

C 6.4092 11.5684 14.2361

O 5.1408 11.7188 14.8267

H 4.9020 10.8913 15.3543

H 6.5763 12.4725 13.6527

H 7.1684 11.5020 14.9694

H 6.4141 10.7544 13.5445

Methanol – cluster with 4 molecules

24

C	3.336406000	9.247856002	16.798721004
O	4.604796002	9.398228002	16.208172004
H	4.843563002	8.570719002	15.680580004

H	3.169269000	10.151942002	17.382176004
H	2.577224000	9.181488002	16.065412004
H	3.331533000	8.433807002	17.490355004
C	3.336406000	13.888956004	16.798721004
O	4.604796002	14.039328004	16.208172004
H	4.843563002	13.211819004	15.680580004
H	3.169269000	14.793042004	17.382176004
H	2.577224000	13.822588004	16.065412004
H	3.331533000	13.074907004	17.490355004
C	6.409194002	6.927306002	14.236129004
O	5.140804002	7.077678002	14.826678004
H	4.902037002	6.250169002	15.354270004
H	6.576331002	7.831392002	13.652674004
H	7.168376002	6.860938002	14.969438004
H	6.414067002	6.113257002	13.544495004
C	6.409194002	11.568406002	14.236129004
O	5.140804002	11.718778002	14.826678004
H	4.902037002	10.891269002	15.354270004
H	6.576331002	12.472492002	13.652674004
H	7.168376002	11.502038002	14.969438004
H	6.414067002	10.754357002	13.544495004

Methanol – cluster with 6 molecules

36

C 3.336406 4.606756 16.798721

O 4.604796 4.757128 16.208172

H 4.843563 3.929619 15.680580

H 3.169269 5.510842 17.382176

H 2.577224 4.540388 16.065412

H 3.331533 3.792707 17.490355

C 3.336406 9.247856 16.798721

O 4.604796 9.398228 16.208172

H 4.843563 8.570719 15.680580

H 3.169269 10.151942 17.382176

H 2.577224 9.181488 16.065412

H 3.331533 8.433807 17.490355

C 3.336406 13.888956 16.798721

O 4.604796 14.039328 16.208172

H 4.843563 13.211819 15.680580

H 3.169269 14.793042 17.382176

H 2.577224 13.822588 16.065412

H 3.331533 13.074907 17.490355

C 6.409194 2.286206 14.236129

O 5.140804 2.436578 14.826678

H 4.902037 1.609069 15.354270

H 6.576331 3.190292 13.652674

H 7.168376 2.219838 14.969438

H 6.414067 1.472157 13.544495

C 6.409194 6.927306 14.236129

O 5.140804 7.077678 14.826678

H 4.902037 6.250169 15.354270

H 6.576331 7.831392 13.652674

H 7.168376 6.860938 14.969438

H 6.414067 6.113257 13.544495

C 6.409194 11.568406 14.236129

O 5.140804 11.718778 14.826678

H 4.902037 10.891269 15.354270

H 6.576331 12.472492 13.652674

H 7.168376 11.502038 14.969438

H 6.414067 10.754357 13.544495

Methanol – cluster with 8 molecules

48

C 0.9000 2.3549 0.9355

O 2.1684 2.2045 1.5260

H 2.4072 3.0320 2.0536

H	0.7329	1.4508	0.3520
H	0.1408	2.4213	1.6688
H	0.8951	3.1689	0.2438
C	0.9000	6.9960	0.9355
O	2.1684	6.8456	1.5260
H	2.4072	7.6731	2.0536
H	0.7329	6.0919	0.3520
H	0.1408	7.0624	1.6688
H	0.8951	7.8100	0.2438
C	0.9000	11.6371	0.9355
O	2.1684	11.4867	1.5260
H	2.4072	12.3142	2.0536
H	0.7329	10.7330	0.3520
H	0.1408	11.7035	1.6688
H	0.8951	12.4511	0.2438
C	0.9000	16.2782	0.9355
O	2.1684	16.1278	1.5260
H	2.4072	16.9553	2.0536
H	0.7329	15.3741	0.3520
H	0.1408	16.3446	1.6688
H	0.8951	17.0922	0.2438
C	3.9728	0.0343	3.4981

O	2.7044	-0.1160	2.9075
H	2.4656	0.7115	2.3799
H	4.1399	-0.8697	4.0815
H	4.7320	0.1007	2.7648
H	3.9777	0.8484	4.1897
C	3.9728	4.6754	3.4981
O	2.7044	4.5251	2.9075
H	2.4656	5.3526	2.3799
H	4.1399	3.7714	4.0815
H	4.7320	4.7418	2.7648
H	3.9777	5.4895	4.1897
C	3.9728	9.3165	3.4981
O	2.7044	9.1662	2.9075
H	2.4656	9.9937	2.3799
H	4.1399	8.4125	4.0815
H	4.7320	9.3829	2.7648
H	3.9777	10.1306	4.1897
C	3.9728	13.9576	3.4981
O	2.7044	13.8073	2.9075
H	2.4656	14.6348	2.3799
H	4.1399	13.0536	4.0815
H	4.7320	14.0240	2.7648

H 3.9777 14.7717 4.1897

Methanol – cluster with 10 molecules

60

C 0.9000 2.3549 0.9355

O 2.1684 2.2045 1.5260

D 2.4072 3.0320 2.0536

D 0.7329 1.4508 0.3520

D 0.1408 2.4213 1.6688

D 0.8951 3.1689 0.2438

C 0.9000 6.9960 0.9355

O 2.1684 6.8456 1.5260

D 2.4072 7.6731 2.0536

D 0.7329 6.0919 0.3520

D 0.1408 7.0624 1.6688

D 0.8951 7.8100 0.2438

C 0.9000 11.6371 0.9355

O 2.1684 11.4867 1.5260

D 2.4072 12.3142 2.0536

D 0.7329 10.7330 0.3520

D 0.1408 11.7035 1.6688

D 0.8951 12.4511 0.2438

C	0.9000	16.2782	0.9355
O	2.1684	16.1278	1.5260
D	2.4072	16.9553	2.0536
D	0.7329	15.3741	0.3520
D	0.1408	16.3446	1.6688
D	0.8951	17.0922	0.2438
C	0.9000	20.9193	0.9355
O	2.1684	20.7689	1.5260
D	2.4072	21.5964	2.0536
D	0.7329	20.0152	0.3520
D	0.1408	20.9857	1.6688
D	0.8951	21.7333	0.2438
C	3.9728	0.0343	3.4981
O	2.7044	-0.1160	2.9075
D	2.4656	0.7115	2.3799
D	4.1399	-0.8697	4.0815
D	4.7320	0.1007	2.7648
D	3.9777	0.8484	4.1897
C	3.9728	4.6754	3.4981
O	2.7044	4.5251	2.9075
D	2.4656	5.3526	2.3799
D	4.1399	3.7714	4.0815

D	4.7320	4.7418	2.7648
D	3.9777	5.4895	4.1897
C	3.9728	9.3165	3.4981
O	2.7044	9.1662	2.9075
D	2.4656	9.9937	2.3799
D	4.1399	8.4125	4.0815
D	4.7320	9.3829	2.7648
D	3.9777	10.1306	4.1897
C	3.9728	13.9576	3.4981
O	2.7044	13.8073	2.9075
D	2.4656	14.6348	2.3799
D	4.1399	13.0536	4.0815
D	4.7320	14.0240	2.7648
D	3.9777	14.7717	4.1897
C	3.9728	18.5987	3.4981
O	2.7044	18.4484	2.9075
D	2.4656	19.2759	2.3799
D	4.1399	17.6947	4.0815
D	4.7320	18.6651	2.7648
D	3.9777	19.4128	4.1897

Methanol – cluster with 12 molecules

72

C 3.336406 4.606756 16.798721

O 4.604796 4.757128 16.208172

H 4.843563 3.929619 15.680580

H 3.169269 5.510842 17.382176

H 2.577224 4.540388 16.065412

H 3.331533 3.792707 17.490355

C 3.336406 9.247856 16.798721

O 4.604796 9.398228 16.208172

H 4.843563 8.570719 15.680580

H 3.169269 10.151942 17.382176

H 2.577224 9.181488 16.065412

H 3.331533 8.433807 17.490355

C 3.336406 13.888956 16.798721

O 4.604796 14.039328 16.208172

H 4.843563 13.211819 15.680580

H 3.169269 14.793042 17.382176

H 2.577224 13.822588 16.065412

H 3.331533 13.074907 17.490355

C 8.209206 4.606756 16.798721

O 9.477596 4.757128 16.208172

H 9.716363 3.929619 15.680580

H 8.042069 5.510842 17.382176

H 7.450024 4.540388 16.065412

H 8.204333 3.792707 17.490355

C 8.209206 9.247856 16.798721

O 9.477596 9.398228 16.208172

H 9.716363 8.570719 15.680580

H 8.042069 10.151942 17.382176

H 7.450024 9.181488 16.065412

H 8.204333 8.433807 17.490355

C 8.209206 13.888956 16.798721

O 9.477596 14.039328 16.208172

H 9.716363 13.211819 15.680580

H 8.042069 14.793042 17.382176

H 7.450024 13.822588 16.065412

H 8.204333 13.074907 17.490355

C 6.409194 2.286206 14.236129

O 5.140804 2.436578 14.826678

H 4.902037 1.609069 15.354270

H 6.576331 3.190292 13.652674

H 7.168376 2.219838 14.969438

H 6.414067 1.472157 13.544495

C 6.409194 6.927306 14.236129

O 5.140804 7.077678 14.826678

H 4.902037 6.250169 15.354270

H 6.576331 7.831392 13.652674

H 7.168376 6.860938 14.969438

H 6.414067 6.113257 13.544495

C 6.409194 11.568406 14.236129

O 5.140804 11.718778 14.826678

H 4.902037 10.891269 15.354270

H 6.576331 12.472492 13.652674

H 7.168376 11.502038 14.969438

H 6.414067 10.754357 13.544495

C 11.281994 2.286206 14.236129

O 10.013604 2.436578 14.826678

H 9.774837 1.609069 15.354270

H 11.449131 3.190292 13.652674

H 12.041176 2.219838 14.969438

H 11.286867 1.472157 13.544495

C 11.281994 6.927306 14.236129

O 10.013604 7.077678 14.826678

H 9.774837 6.250169 15.354270

H 11.449131 7.831392 13.652674

H 12.041176 6.860938 14.969438

H 11.286867 6.113257 13.544495

C 11.281994 11.568406 14.236129

O 10.013604 11.718778 14.826678

H 9.774837 10.891269 15.354270

H 11.449131 12.472492 13.652674

H 12.041176 11.502038 14.969438

H 11.286867 10.754357 13.544495

Coordinates of Ethanol clusters

Ethanol – cluster with 2 molecules (Fig. 2a upper dimer in the Article)

18

C 3.4232 8.9377 9.2482

C 4.6868 8.7890 8.4300

O 3.1964 7.8104 10.0857

H 3.0890 7.1848 9.5128

H 3.3615 9.6623 9.8194

H 2.6408 9.0498 8.5769

H 4.6415 7.9762 7.7539

H 5.4593 8.5819 9.0449

H 4.9275 9.5660 7.9233

C 1.4659 5.2902 8.2437

C 0.7981 5.1381 9.5766

O 2.8345 5.6536 8.4518

H 3.1610 5.7396 7.7378

H 1.0257 5.8979 7.6409

H 1.4236 4.5284 7.6409

H 1.2139 4.4320 10.0534

H 0.8075 5.9805 10.1422

H -0.1139 4.9482 9.5048

Ethanol – cluster with 2 molecules (Fig. 2a lower dimer in the Article)

18

C 3.4232 8.9377 9.2482

C 4.6868 8.7890 8.4300

O 3.1964 7.8104 10.0857

H 3.0890 7.1848 9.5128

H 3.3615 9.6623 9.8194

H 2.6408 9.0498 8.5769

H 4.6415 7.9762 7.7539

H 5.4593 8.5819 9.0449

H 4.9275 9.5660 7.9233

C 0.5937 8.4738 12.2779

C -0.0741 8.6259 13.6109

O 1.9623 8.1104 12.4861

H 2.2887 8.0244 11.7720

H 0.1535 7.8661 11.6752

H 0.5514 9.2356 11.6752

H 0.3416 9.3320 14.0877

H -0.0647 7.7835 14.1765

H -0.9862 8.8158 13.5391

Ethanol – cluster with 4 molecules

36

C	3.423174000	8.937653002	9.248193002
C	4.686757002	8.789002002	8.430040002
O	3.196392000	7.810382002	10.085710002
H	3.089040000	7.184808002	9.512842002
H	3.361500000	9.662328002	9.819448002
H	2.640783000	9.049830002	8.576888002
H	4.641524002	7.976238002	7.753894002
H	5.459314002	8.581854002	9.044865002
H	4.927510002	9.565980002	7.923334002
C	4.295418002	4.826347002	5.213909002
C	5.559000002	4.974998002	4.395756002
O	4.068635000	5.953618002	6.051426002
H	3.961283000	6.579192002	5.478558002

H	4.233743002	4.101672000	5.785163002
H	3.513026000	4.714170002	4.542604002
H	5.513767002	5.787762002	3.719610000
H	6.331557002	5.182146002	5.010581002
H	5.799753002	4.198020002	3.889050000
C	0.593661000	8.473807002	12.277940002
C	-0.074144000	8.625899002	13.610868004
O	1.962267000	8.110437002	12.486109002
H	2.288718000	8.024412002	11.772041002
H	0.153474000	7.866126002	11.675218002
H	0.551372000	9.235644002	11.675218002
H	0.341637000	9.331992002	14.087720004
H	-0.064696000	7.783542002	14.176474004
H	-0.986151000	8.815842002	13.539057004
C	1.465904000	5.290193002	8.243656002
C	0.798099000	5.138101002	9.576584002
O	2.834510000	5.653563002	8.451825002
H	3.160961000	5.739588002	7.737757002
H	1.025718000	5.897874002	7.640934002
H	1.423616000	4.528356002	7.640934002
H	1.213880000	4.432008002	10.053436002
H	0.807547000	5.980458002	10.142190002

H -0.113908000 4.948158002 9.504773002

Ethanol – cluster with 8 molecules (Fig. 2b in the Article)

72

C 5.167661 8.937653 1.179625

C 6.431243 8.789002 0.361472

O 4.940878 7.810382 2.017142

H 4.833526 7.184808 1.444274

H 5.105986 9.662328 1.750879

H 4.385269 9.049830 0.508320

H 6.386010 7.976238 -0.314674

H 7.203800 8.581854 0.976297

H 6.671996 9.565980 -0.145234

C 3.423174 8.937653 9.248193

C 4.686757 8.789002 8.430040

O 3.196392 7.810382 10.085710

H 3.089040 7.184808 9.512842

H 3.361500 9.662328 9.819448

H 2.640783 9.049830 8.576888

H 4.641524 7.976238 7.753894

H 5.459314 8.581854 9.044865

H 4.927510 9.565980 7.923334

C 4.295418 4.826347 5.213909

C 5.559000 4.974998 4.395756

O 4.068635 5.953618 6.051426

H 3.961283 6.579192 5.478558

H 4.233743 4.101672 5.785163

H 3.513026 4.714170 4.542604

H 5.513767 5.787762 3.719610

H 6.331557 5.182146 5.010581

H 5.799753 4.198020 3.889050

C 2.550931 4.826347 13.282477

C 3.814514 4.974998 12.464324

O 2.324149 5.953618 14.119994

H 2.216797 6.579192 13.547126

H 2.489257 4.101672 13.853732

H 1.768540 4.714170 12.611172

H 3.769281 5.787762 11.788178

H 4.587071 5.182146 13.079149

H 4.055266 4.198020 11.957618

C 2.338147 8.473807 4.209372

C 1.670342 8.625899 5.542300

O 3.706753 8.110437 4.417541

H 4.033204 8.024412 3.703473

H 1.897961 7.866126 3.606650

H 2.295859 9.235644 3.606650

H 2.086123 9.331992 6.019152

H 1.679790 7.783542 6.107906

H 0.758335 8.815842 5.470489

C 0.593661 8.473807 12.277940

C -0.074144 8.625899 13.610868

O 1.962267 8.110437 12.486109

H 2.288718 8.024412 11.772041

H 0.153474 7.866126 11.675218

H 0.551372 9.235644 11.675218

H 0.341637 9.331992 14.087720

H -0.064696 7.783542 14.176474

H -0.986151 8.815842 13.539057

C 3.210390 5.290193 0.175088

C 2.542585 5.138101 1.508015

O 4.578996 5.653563 0.383257

H 4.905447 5.739588 -0.330811

H 2.770204 5.897874 -0.427634

H 3.168102 4.528356 -0.427634

H 2.958366 4.432008 1.984868

H 2.552033 5.980458 2.073622

H 1.630578 4.948158 1.436205

C 1.465904 5.290193 8.243656

C 0.798099 5.138101 9.576584

O 2.834510 5.653563 8.451825

H 3.160961 5.739588 7.737757

H 1.025718 5.897874 7.640934

H 1.423616 4.528356 7.640934

H 1.213880 4.432008 10.053436

H 0.807547 5.980458 10.142190

H -0.113908 4.948158 9.504773

Ethanol – cluster with 10 molecules

90

C 5.1677 15.8197 1.1796

C 6.4312 15.6710 0.3615

H 4.8335 14.0668 1.4443

H 5.1060 16.5443 1.7509

H 4.3853 15.9318 0.5083

H 6.3860 14.8582 -0.3147

H 7.2038 15.4639 0.9763

H 6.6720 16.4480 -0.1452

O 4.9409 14.6924 2.0171

C	3.4232	15.8197	9.2482
C	4.6868	15.6710	8.4300
H	3.0890	14.0668	9.5128
H	3.3615	16.5443	9.8194
H	2.6408	15.9318	8.5769
H	4.6415	14.8582	7.7539
H	5.4593	15.4639	9.0449
H	4.9275	16.4480	7.9233
O	3.1964	14.6924	10.0857
C	1.6787	15.8197	17.3168
C	2.9423	15.6710	16.4986
H	1.3446	14.0668	17.5814
H	1.6170	16.5443	17.8880
H	0.8963	15.9318	16.6455
H	2.8970	14.8582	15.8225
H	3.7148	15.4639	17.1134
H	3.1830	16.4480	15.9919
O	1.4519	14.6924	18.1543
C	4.2954	11.7083	5.2139
C	5.5590	11.8570	4.3958
H	3.9613	13.4612	5.4786
H	4.2337	10.9837	5.7852

H	3.5130	11.5962	4.5426
H	5.5138	12.6698	3.7196
H	6.3316	12.0641	5.0106
H	5.7998	11.0800	3.8891
O	4.0686	12.8356	6.0514
C	2.5509	11.7083	13.2825
C	3.8145	11.8570	12.4643
H	2.2168	13.4612	13.5471
H	2.4893	10.9837	13.8537
H	1.7685	11.5962	12.6112
H	3.7693	12.6698	11.7882
H	4.5871	12.0641	13.0791
H	4.0553	11.0800	11.9576
O	2.3241	12.8356	14.1200
C	2.3381	15.3558	4.2094
C	1.6703	15.5079	5.5423
H	4.0332	14.9064	3.7035
H	1.8980	14.7481	3.6067
H	2.2959	16.1176	3.6067
H	2.0861	16.2140	6.0192
H	1.6798	14.6655	6.1079
H	0.7583	15.6978	5.4705

O 3.7068 14.9924 4.4175

C 0.5937 15.3558 12.2779

C -0.0741 15.5079 13.6109

H 2.2887 14.9064 11.7720

H 0.1535 14.7481 11.6752

H 0.5514 16.1176 11.6752

H 0.3416 16.2140 14.0877

H -0.0647 14.6655 14.1765

H -0.9862 15.6978 13.5391

O 1.9623 14.9924 12.4861

C 3.2104 12.1722 0.1751

C 2.5426 12.0201 1.5080

H 4.9054 12.6216 -0.3308

H 2.7702 12.7799 -0.4276

H 3.1681 11.4104 -0.4276

H 2.9584 11.3140 1.9849

H 2.5520 12.8625 2.0736

H 1.6306 11.8302 1.4362

O 4.5790 12.5356 0.3833

C 1.4659 12.1722 8.2437

C 0.7981 12.0201 9.5766

H 3.1610 12.6216 7.7378

H 1.0257 12.7799 7.6409

H 1.4236 11.4104 7.6409

H 1.2139 11.3140 10.0534

H 0.8075 12.8625 10.1422

H -0.1139 11.8302 9.5048

O 2.8345 12.5356 8.4518

C -0.2786 12.1722 16.3122

C -0.9464 12.0201 17.6452

H 1.4165 12.6216 15.8063

H -0.7188 12.7799 15.7095

H -0.3209 11.4104 15.7095

H -0.5306 11.3140 18.1220

H -0.9369 12.8625 18.2108

H -1.8584 11.8302 17.5733

O 1.0900 12.5356 16.5204

Ethanol – cluster with 12 molecules

108

C 5.1677 15.8197 1.1796

C 6.4312 15.6710 0.3615

H 4.8335 14.0668 1.4443

H 5.1060 16.5443 1.7509

H	4.3853	15.9318	0.5083
H	6.3860	14.8582	-0.3147
H	7.2038	15.4639	0.9763
H	6.6720	16.4480	-0.1452
O	4.9409	14.6924	2.0171
C	3.4232	15.8197	9.2482
C	4.6868	15.6710	8.4300
H	3.0890	14.0668	9.5128
H	3.3615	16.5443	9.8194
H	2.6408	15.9318	8.5769
H	4.6415	14.8582	7.7539
H	5.4593	15.4639	9.0449
H	4.9275	16.4480	7.9233
O	3.1964	14.6924	10.0857
C	1.6787	15.8197	17.3168
C	2.9423	15.6710	16.4986
H	1.3446	14.0668	17.5814
H	1.6170	16.5443	17.8880
H	0.8963	15.9318	16.6455
H	2.8970	14.8582	15.8225
H	3.7148	15.4639	17.1134
H	3.1830	16.4480	15.9919

O	1.4519	14.6924	18.1543
C	4.2954	11.7083	5.2139
C	5.5590	11.8570	4.3958
H	3.9613	13.4612	5.4786
H	4.2337	10.9837	5.7852
H	3.5130	11.5962	4.5426
H	5.5138	12.6698	3.7196
H	6.3316	12.0641	5.0106
H	5.7998	11.0800	3.8891
O	4.0686	12.8356	6.0514
C	2.5509	11.7083	13.2825
C	3.8145	11.8570	12.4643
H	2.2168	13.4612	13.5471
H	2.4893	10.9837	13.8537
H	1.7685	11.5962	12.6112
H	3.7693	12.6698	11.7882
H	4.5871	12.0641	13.0791
H	4.0553	11.0800	11.9576
O	2.3241	12.8356	14.1200
C	0.8064	11.7083	21.3510
C	2.0700	11.8570	20.5329
H	0.4723	13.4612	21.6157

H 0.7448 10.9837 21.9223

H 0.0241 11.5962 20.6797

H 2.0248 12.6698 19.8567

H 2.8426 12.0641 21.1477

H 2.3108 11.0800 20.0262

O 0.5797 12.8356 22.1886

C 2.3381 15.3558 4.2094

C 1.6703 15.5079 5.5423

H 4.0332 14.9064 3.7035

H 1.8980 14.7481 3.6067

H 2.2959 16.1176 3.6067

H 2.0861 16.2140 6.0192

H 1.6798 14.6655 6.1079

H 0.7583 15.6978 5.4705

O 3.7068 14.9924 4.4175

C 0.5937 15.3558 12.2779

C -0.0741 15.5079 13.6109

H 2.2887 14.9064 11.7720

H 0.1535 14.7481 11.6752

H 0.5514 16.1176 11.6752

H 0.3416 16.2140 14.0877

H -0.0647 14.6655 14.1765

H -0.9862 15.6978 13.5391

O 1.9623 14.9924 12.4861

C -1.1508 15.3558 20.3465

C -1.8186 15.5079 21.6794

H 0.5442 14.9064 19.8406

H -1.5910 14.7481 19.7438

H -1.1931 16.1176 19.7438

H -1.4028 16.2140 22.1563

H -1.8092 14.6655 22.2450

H -2.7306 15.6978 21.6076

O 0.2178 14.9924 20.5547

C 3.2104 12.1722 0.1751

C 2.5426 12.0201 1.5080

H 4.9054 12.6216 -0.3308

H 2.7702 12.7799 -0.4276

H 3.1681 11.4104 -0.4276

H 2.9584 11.3140 1.9849

H 2.5520 12.8625 2.0736

H 1.6306 11.8302 1.4362

O 4.5790 12.5356 0.3833

C 1.4659 12.1722 8.2437

C 0.7981 12.0201 9.5766

H 3.1610 12.6216 7.7378

H 1.0257 12.7799 7.6409

H 1.4236 11.4104 7.6409

H 1.2139 11.3140 10.0534

H 0.8075 12.8625 10.1422

H -0.1139 11.8302 9.5048

O 2.8345 12.5356 8.4518

C -0.2786 12.1722 16.3122

C -0.9464 12.0201 17.6452

H 1.4165 12.6216 15.8063

H -0.7188 12.7799 15.7095

H -0.3209 11.4104 15.7095

H -0.5306 11.3140 18.1220

H -0.9369 12.8625 18.2108

H -1.8584 11.8302 17.5733

O 1.0900 12.5356 16.5204

Coordinates of Acetonitrile clusters

Acetonitrile – cluster with 2 molecules. optimized dimer (Fig. 4a in the Article)

12

C 3.87E-02 -1.401853782 6.72E-02

C -3.32E-02 4.62E-02 -5.72E-02

N -9.56E-02 1.183855554 -0.165503781

H 1.078033955 -1.713442991 9.56E-02

H -0.455898096 -1.713412076 0.981698478

H -0.456499147 -1.848374611 -0.790142996

C -1.767542851 1.620453416 -3.061513101

C -1.695689386 0.172426119 -2.937136428

N -1.633260404 -0.965258582 -2.828850827

H -1.272922761 1.932005285 -3.976046386

H -2.806855063 1.932054795 -3.089943993

H -1.27232241 2.066974361 -2.204205939

Acetonitrile – cluster with 2 molecules. dimer from the crystal (Fig. 4b in the Article)

12

C 1.2806 12.4990 0.6353

C 1.7426 11.3952 1.4418

N 2.0972 10.5283 2.0774

H 1.1809 12.2745 -0.3234

H 1.9191 13.1705 0.5960

H 0.4550 12.8764 0.9838

C 1.8708 11.8759 5.3322

C 1.4088 12.9797 4.5257

N 1.0542 13.8466 3.8902

H 1.9705 12.1004 6.2909

H 1.2322 11.2044 5.3714

H 2.6964 11.4984 4.9837

Acetonitrile – cluster with 4 molecules

24

C 0.032200 12.056000 1.954400

C 0.692400 11.167800 2.880500

N 1.204500 10.473100 3.612800

H -0.518400 11.586300 1.279400

H 0.649000 12.487700 1.412800

H -0.497100 12.727500 2.417500

C 4.142200 12.056000 1.954400

C 4.802400 11.167800 2.880500

N 5.314500 10.473100 3.612800

H 3.591600 11.586300 1.279400

H 4.759000 12.487700 1.412800

H 3.612900 12.727500 2.417500

C 2.637300 12.754000 5.894500

C 1.977100 13.642200 4.968400

N 1.465000 14.336900 4.236100

H 3.187900 13.223700 6.569500

H 2.020400 12.322300 6.436100

H 3.166600 12.082500 5.431400

C 6.747300 12.754000 5.894500

C 6.087100 13.642200 4.968400

N 5.575000 14.336900 4.236100

H 7.297900 13.223700 6.569500

H 6.130400 12.322300 6.436100

H 7.276600 12.082500 5.431400

Acetonitrile – cluster with 8 molecules (Fig. 4c in the Article)

48

C -0.9641 24.4610 25.5168

C -1.6243 23.5728 24.5906

N -2.1364 22.8781 23.8583

H -0.4135 23.9913 26.1918

H -1.5809 24.8927 26.0583

H -0.4348 25.1325 25.0537

C 3.1459 24.4610 25.5168

C 2.4857 23.5728 24.5906

N 1.9736 22.8781 23.8583

H 3.6965 23.9913 26.1918

H 2.5291 24.8927 26.0583

H 3.6752 25.1325 25.0537

C 7.2559 24.4610 25.5168

C 6.5957 23.5728 24.5906

N 6.0836 22.8781 23.8583

H 7.8065 23.9913 26.1918

H 6.6391 24.8927 26.0583

H 7.7852 25.1325 25.0537

C 11.3659 24.4610 25.5168

C 10.7057 23.5728 24.5906

N 10.1936 22.8781 23.8583

H 11.9165 23.9913 26.1918

H 10.7491 24.8927 26.0583

H 11.8952 25.1325 25.0537

C -3.5692 25.1590 21.5766

C -2.9090 26.0472 22.5028

N -2.3969 26.7419 23.2351

H -4.1198 25.6287 20.9016

H -2.9523 24.7273 21.0351

H -4.0985 24.4875 22.0397

C 0.5408 25.1590 21.5766

C 1.2010 26.0472 22.5028

N 1.7131 26.7419 23.2351

H -0.0098 25.6287 20.9016

H 1.1577 24.7273 21.0351

H 0.0115 24.4875 22.0397

C 4.6508 25.1590 21.5766

C 5.3110 26.0472 22.5028

N 5.8231 26.7419 23.2351

H 4.1002 25.6287 20.9016

H 5.2677 24.7273 21.0351

H 4.1215 24.4875 22.0397

C 8.7608 25.1590 21.5766

C 9.4210 26.0472 22.5028

N 9.9331 26.7419 23.2351

H 8.2102 25.6287 20.9016

H 9.3777 24.7273 21.0351

H 8.2315 24.4875 22.0397

Acetonitrile – cluster with 8 molecules (Fig. 4d in the Article)

48

C -1.408376997 12.05600599 9.803276971

C -0.748141 11.16780797 10.72944699

N -0.236041 10.473128 11.46174898

H -1.958940998 11.58626999 9.128271013

H	-0.791530001	12.48769997	9.261701987
H	-1.937659999	12.72753001	10.26636201
C	2.701622995	12.05600599	9.803276971
C	3.361858998	11.16780797	10.72944699
N	3.873958996	10.473128	11.46174898
H	2.151059	11.58626999	9.128271013
H	3.318469996	12.48769997	9.261701987
H	2.172339999	12.72753001	10.26636201
C	1.917019997	16.19100601	9.818974007
C	1.256784	15.30280799	8.892803983
N	0.744684002	14.60812797	8.160501998
H	2.467583997	15.72127	10.49397997
H	1.300174001	16.62269999	10.36054798
H	2.446302998	16.86252998	9.355888969
C	6.027019995	16.19100601	9.818974007
C	5.366784008	15.30280799	8.892803983
N	4.854683994	14.60812797	8.160501998
H	6.577584005	15.72127	10.49397997
H	5.410173999	16.62269999	10.36054798
H	6.556302985	16.86252998	9.355888969
C	1.196748999	12.75399401	13.74342399
C	0.536513001	13.64219197	12.81725397

N	2.44E-02	14.33687199	12.08495198
H	1.747312999	13.22373001	14.41843
H	0.579901997	12.32229997	14.28499897
H	1.726032	12.08246998	13.28033901
C	5.306749012	12.75399401	13.74342399
C	4.646512993	13.64219197	12.81725397
N	4.134411995	14.33687199	12.08495198
H	5.85731297	13.22373001	14.41843
H	4.689901995	12.32229997	14.28499897
H	5.836032003	12.08246998	13.28033901
C	-2.128647996	8.61899399	13.72772701
C	-1.468411998	9.507192004	14.65389698
N	-0.956312	10.20187198	15.38619896
H	-2.679212996	9.088729992	13.052721
H	-1.511802	8.187300008	13.18615298
H	-2.657930997	7.947470018	14.19081199
C	1.981351996	8.61899399	13.72772701
C	2.641587999	9.507192004	14.65389698
N	3.153687997	10.20187198	15.38619896
H	1.430786996	9.088729992	13.052721
H	2.598197998	8.187300008	13.18615298
H	1.452069	7.947470018	14.19081199

Coordinates of Pyridine clusters

Pyridine – cluster with 2 molecules. optimized dimer (Fig. 6a in the Article)

22

C -1.420929488 -1.321800807 -1.15965991

C -1.781594633 -0.279807071 -0.322953609

C -0.986650783 0.851585164 -0.295904767

C 0.138432387 0.886731701 -1.103861518

N 0.500821524 -0.107168499 -1.908002881

C -0.275307972 -1.185727037 -1.927667492

H -2.009325388 -2.223402424 -1.218238409

H -2.657248951 -0.35079435 0.303027954

H -1.221107036 1.685222425 0.345712366

H 0.782364245 1.754973479 -1.099578299

H 3.32E-02 -1.982201215 -2.59039483

C 1.510016355 -1.202079099 1.422333395

C 2.214612325 -1.98E-02 1.559261432

C 1.718444779 0.951797076 2.411051483

C 0.534903704 0.69623115 3.085477224

N -0.153894668 -0.433349315 2.962116587

C 0.33734114 -1.357956056 2.143649302

H 1.850458442 -1.983354932 0.762580505

H	3.127035236	0.142832237	1.007073948
H	2.232262761	1.889022178	2.552869006
H	0.121307674	1.435284971	3.757442308
H	-0.237810045	-2.26861279	2.053386196

Pyridine – cluster with 4 molecules (Fig. 6b in the Article)

44

N	1.973472	3.911408	1.351883
C	0.991589	3.950202	0.477016
H	1.016392	4.611746	-0.175672
C	-0.067939	3.075631	0.466543
H	-0.738165	3.153220	-0.173419
C	-0.122938	2.077191	1.425417
H	-0.827672	1.471457	1.451543
C	0.901003	2.015937	2.332941
H	0.905317	1.357797	2.989796
C	1.915778	2.927261	2.266164
H	2.605414	2.863965	2.887320
N	0.722528	2.894592	6.982383
C	1.704411	2.855798	6.107516
H	1.679608	2.194254	5.454828
C	2.763939	3.730369	6.097043

H	3.434165	3.652780	5.457081
C	2.818938	4.728809	7.055917
H	3.523672	5.334543	7.082043
C	1.794997	4.790063	7.963441
H	1.790683	5.448203	8.620296
C	0.780222	3.878739	7.896664
H	0.090586	3.942035	8.517820
N	3.418528	7.314408	4.278617
C	4.400411	7.353202	5.153484
H	4.375608	8.014746	5.806172
C	5.459939	6.478631	5.163957
H	6.130165	6.556220	5.803919
C	5.514938	5.480191	4.205083
H	6.219672	4.874457	4.178957
C	4.490997	5.418937	3.297559
H	4.486683	4.760797	2.640704
C	3.476222	6.330261	3.364336
H	2.786586	6.266965	2.743180
N	4.669472	-0.508408	9.909117
C	3.687589	-0.547202	10.783984
H	3.712392	-1.208746	11.436672
C	2.628061	0.327369	10.794457

H	1.957835	0.249780	11.434419
C	2.573062	1.325809	9.835583
H	1.868328	1.931543	9.809457
C	3.597003	1.387063	8.928059
H	3.601317	2.045203	8.271204
C	4.611778	0.475739	8.994836
H	5.301414	0.539035	8.373680

Pyridine – cluster with 6 molecules (Fig. 6c in the Article)

66

N	1.973472000	3.911408000	1.351883000
C	0.991589000	3.950202000	0.477016000
C	-0.067939000	3.075631000	0.466543000
C	-0.122938000	2.077191000	1.425417000
C	0.901003000	2.015937000	2.332941000
C	1.915778000	2.927261000	2.266164000
H	1.016392000	4.611746001	-0.175672000
H	-0.738165000	3.153220000	-0.173419000
H	-0.827672000	1.471457000	1.451543000
H	0.905317000	1.357797000	2.989796000
H	2.605414000	2.863965000	2.887320000
N	7.365472001	3.911408000	1.351883000

C	6.383589001	3.950202000	0.477016000
C	5.324061001	3.075631000	0.466543000
C	5.269062001	2.077191000	1.425417000
C	6.293003001	2.015937000	2.332941000
C	7.307778001	2.927261000	2.266164000
H	6.408392001	4.611746001	-0.175672000
H	4.653835001	3.153220000	-0.173419000
H	4.564328001	1.471457000	1.451543000
H	6.297317001	1.357797000	2.989796000
H	7.997414001	2.863965000	2.887320000
N	12.757472002	3.911408000	1.351883000
C	11.775589001	3.950202000	0.477016000
C	10.716061001	3.075631000	0.466543000
C	10.661062001	2.077191000	1.425417000
C	11.685003001	2.015937000	2.332941000
C	12.699778002	2.927261000	2.266164000
H	11.800392001	4.611746001	-0.175672000
H	10.045835001	3.153220000	-0.173419000
H	9.956328001	1.471457000	1.451543000
H	11.689317001	1.357797000	2.989796000
H	13.389414002	2.863965000	2.887320000
N	3.418528000	7.314408001	4.278617001

C	4.400411001	7.353202001	5.153484001
C	5.459939001	6.478631001	5.163957001
C	5.514938001	5.480191001	4.205083001
C	4.490997001	5.418937001	3.297559000
C	3.476222000	6.330261001	3.364336000
H	4.375608001	8.014746001	5.806172001
H	6.130165001	6.556220001	5.803919001
H	6.219672001	4.874457001	4.178957000
H	4.486683001	4.760797001	2.640704000
H	2.786586000	6.266965001	2.743180000
N	8.810528001	7.314408001	4.278617001
C	9.792411001	7.353202001	5.153484001
C	10.851939001	6.478631001	5.163957001
C	10.906938001	5.480191001	4.205083001
C	9.882997001	5.418937001	3.297559000
C	8.868222001	6.330261001	3.364336000
H	9.767608001	8.014746001	5.806172001
H	11.522165001	6.556220001	5.803919001
H	11.611672001	4.874457001	4.178957000
H	9.878683001	4.760797001	2.640704000
H	8.178586001	6.266965001	2.743180000
N	14.202528002	7.314408001	4.278617001

C	15.184411002	7.353202001	5.153484001
C	16.243939002	6.478631001	5.163957001
C	16.298938002	5.480191001	4.205083001
C	15.274997002	5.418937001	3.297559000
C	14.260222002	6.330261001	3.364336000
H	15.159608002	8.014746001	5.806172001
H	16.914165002	6.556220001	5.803919001
H	17.003672002	4.874457001	4.178957000
H	15.270683002	4.760797001	2.640704000
H	13.570586002	6.266965001	2.743180000

Pyridine – cluster with 6 molecules (Fig. 6d in the Article)

66

N	1.973472000	3.911408000	1.351883000
C	0.991589000	3.950202000	0.477016000
C	-0.067939000	3.075631000	0.466543000
C	-0.122938000	2.077191000	1.425417000
C	0.901003000	2.015937000	2.332941000
C	1.915778000	2.927261000	2.266164000
H	1.016392000	4.611746001	-0.175672000
H	-0.738165000	3.153220000	-0.173419000
H	-0.827672000	1.471457000	1.451543000

H	0.905317000	1.357797000	2.989796000
H	2.605414000	2.863965000	2.887320000
N	7.365472001	3.911408000	1.351883000
C	6.383589001	3.950202000	0.477016000
C	5.324061001	3.075631000	0.466543000
C	5.269062001	2.077191000	1.425417000
C	6.293003001	2.015937000	2.332941000
C	7.307778001	2.927261000	2.266164000
H	6.408392001	4.611746001	-0.175672000
H	4.653835001	3.153220000	-0.173419000
H	4.564328001	1.471457000	1.451543000
H	6.297317001	1.357797000	2.989796000
H	7.997414001	2.863965000	2.887320000
N	12.757472002	3.911408000	1.351883000
C	11.775589001	3.950202000	0.477016000
C	10.716061001	3.075631000	0.466543000
C	10.661062001	2.077191000	1.425417000
C	11.685003001	2.015937000	2.332941000
C	12.699778002	2.927261000	2.266164000
H	11.800392001	4.611746001	-0.175672000
H	10.045835001	3.153220000	-0.173419000
H	9.956328001	1.471457000	1.451543000

H	11.689317001	1.357797000	2.989796000
H	13.389414002	2.863965000	2.887320000
N	3.418528000	7.314408001	4.278617001
C	4.400411001	7.353202001	5.153484001
C	5.459939001	6.478631001	5.163957001
C	5.514938001	5.480191001	4.205083001
C	4.490997001	5.418937001	3.297559000
C	3.476222000	6.330261001	3.364336000
H	4.375608001	8.014746001	5.806172001
H	6.130165001	6.556220001	5.803919001
H	6.219672001	4.874457001	4.178957000
H	4.486683001	4.760797001	2.640704000
H	2.786586000	6.266965001	2.743180000
N	8.810528001	7.314408001	4.278617001
C	9.792411001	7.353202001	5.153484001
C	10.851939001	6.478631001	5.163957001
C	10.906938001	5.480191001	4.205083001
C	9.882997001	5.418937001	3.297559000
C	8.868222001	6.330261001	3.364336000
H	9.767608001	8.014746001	5.806172001
H	11.522165001	6.556220001	5.803919001
H	11.611672001	4.874457001	4.178957000

H	9.878683001	4.760797001	2.640704000
H	8.178586001	6.266965001	2.743180000
N	14.202528002	7.314408001	4.278617001
C	15.184411002	7.353202001	5.153484001
C	16.243939002	6.478631001	5.163957001
C	16.298938002	5.480191001	4.205083001
C	15.274997002	5.418937001	3.297559000
C	14.260222002	6.330261001	3.364336000
H	15.159608002	8.014746001	5.806172001
H	16.914165002	6.556220001	5.803919001
H	17.003672002	4.874457001	4.178957000
H	15.270683002	4.760797001	2.640704000
H	13.570586002	6.266965001	2.743180000

Coordinates of Acetone clusters

Acetone – cluster with 2 molecules. optimized dimer (Fig. 8a in the Article)

20

C 0.162239 0.001772 0.389792

O 0.151321 0.001761 1.600582

C 0.184517 1.279138 -0.400099

C 0.202593 -1.275275 -0.399874

H 0.095003 -2.124103 0.265554

H -0.580356 -1.282049 -1.155381

H 1.162861 -1.323041 -0.912268

H 0.070088 2.126815 0.265652

H 1.141391 1.338112 -0.917555

H -0.602304 1.276625 -1.151642

C 3.185776 0.019718 1.328150

O 3.198192 0.019327 0.117378

C 3.143941 1.296980 2.117414

C 3.163141 -1.257338 2.118550

H 3.950154 -1.254649 2.869908

H 2.206391 -1.315780 2.636260

H 3.277242 -2.105356 1.453192

H 3.925512 1.303997 2.874344

H 3.252569 2.145687 1.452013

H 2.182725 1.344657 2.628029

Acetone – cluster with 2 molecules. dimer from the crystal (Fig. 8b in the Article)

20

O 8.2853 7.0359 18.7827

C 8.0501 5.8531 18.7827

C 7.8756 5.0721 17.5086

C 7.8756 5.0721 20.0569

H 8.1952 5.5614 16.7918

H 6.9870 4.8349 17.4057

H 8.4381 4.3113 17.5270

H 8.1952 5.5614 20.7737

H 6.9870 4.8349 20.1598

H 8.4381 4.3113 20.0385

O 4.4997 9.7071 18.7827

C 4.7349 8.5243 18.7827

C 4.9094 7.7433 20.0569

C 4.9094 7.7433 17.5086

H 4.5898 8.2326 20.7737

H 5.7980 7.5061 20.1598

H 4.3469 6.9825 20.0385

H 4.5898 8.2326 16.7918

H 5.7980 7.5061 17.4057

H 4.3469 6.9825 17.5270

Acetone – cluster with 4 molecules (Fig. 8c in the Article)

40

O 1.892819000 12.378341001 18.782750002

C 1.657575000 11.195533001 18.782750002

C 1.483060000 10.414475001 17.508636002

C	1.483060000	10.414475001	20.056864002
H	1.802685000	10.903838001	16.791778002
H	0.594503000	10.177272001	17.405706002
H	2.045600000	9.653717001	17.526989002
H	1.802685000	10.903838001	20.773722002
H	0.594503000	10.177272001	20.159794002
H	2.045600000	9.653717001	20.038511002
O	8.285319001	7.035941001	18.782750002
C	8.050075001	5.853133001	18.782750002
C	7.875560001	5.072075001	17.508636002
C	7.875560001	5.072075001	20.056864002
H	8.195185001	5.561438001	16.791778002
H	6.987003001	4.834872001	17.405706002
H	8.438100001	4.311317001	17.526989002
H	8.195185001	5.561438001	20.773722002
H	6.987003001	4.834872001	20.159794002
H	8.438100001	4.311317001	20.038511002
O	4.499681001	9.707141001	18.782750002
C	4.734925001	8.524333001	18.782750002
C	4.909440001	7.743275001	20.056864002
C	4.909440001	7.743275001	17.508636002
H	4.589815001	8.232638001	20.773722002

H	5.797998001	7.506072001	20.159794002
H	4.346900001	6.982517001	20.038511002
H	4.589815001	8.232638001	16.791778002
H	5.797998001	7.506072001	17.405706002
H	4.346900001	6.982517001	17.526989002
O	10.892181001	4.364741001	18.782750002
C	11.127425001	3.181933000	18.782750002
C	11.301940001	2.400875000	20.056864002
C	11.301940001	2.400875000	17.508636002
H	10.982315001	2.890238000	20.773722002
H	12.190498001	2.163672000	20.159794002
H	10.739400001	1.640117000	20.038511002
H	10.982315001	2.890238000	16.791778002
H	12.190498001	2.163672000	17.405706002
H	10.739400001	1.640117000	17.526989002

Acetone – cluster with 8 molecules (Fig. 8d in the Article)

80

O 1.8928 0.9777 24.1493

C 1.6576 2.1605 24.1493

C 1.4831 2.9415 22.8751

H 1.8027 2.4522 22.1583

H	0.5945	3.1787	22.7722
H	2.0456	3.7023	22.8935
C	1.4831	2.9415	25.4234
H	1.8027	2.4522	26.1402
H	0.5945	3.1787	25.5263
H	2.0456	3.7023	25.4050
O	8.2853	0.9777	24.1493
C	8.0501	2.1605	24.1493
C	7.8756	2.9415	22.8751
H	8.1952	2.4522	22.1583
H	6.9870	3.1787	22.7722
H	8.4381	3.7023	22.8935
C	7.8756	2.9415	25.4234
H	8.1952	2.4522	26.1402
H	6.9870	3.1787	25.5263
H	8.4381	3.7023	25.4050
O	14.6778	0.9777	24.1493
C	14.4426	2.1605	24.1493
C	14.2681	2.9415	22.8751
H	14.5877	2.4522	22.1583
H	13.3795	3.1787	22.7722
H	14.8306	3.7023	22.8935

C 14.2681 2.9415 25.4234

H 14.5877 2.4522 26.1402

H 13.3795 3.1787 25.5263

H 14.8306 3.7023 25.4050

O 4.4997 -1.6935 24.1493

C 4.7349 -0.5107 24.1493

C 4.9094 0.2703 22.8751

H 4.5898 -0.2190 22.1583

H 5.7980 0.5075 22.7722

H 4.3469 1.0311 22.8935

C 4.9094 0.2703 25.4234

H 4.5898 -0.2190 26.1402

H 5.7980 0.5075 25.5263

H 4.3469 1.0311 25.4050

O 4.4997 3.6489 24.1493

C 4.7349 4.8317 24.1493

C 4.9094 5.6127 22.8751

H 4.5898 5.1234 22.1583

H 5.7980 5.8499 22.7722

H 4.3469 6.3735 22.8935

C 4.9094 5.6127 25.4234

H 4.5898 5.1234 26.1402

H 5.7980 5.8499 25.5263

H 4.3469 6.3735 25.4050

O 10.8922 -1.6935 24.1493

C 11.1274 -0.5107 24.1493

C 11.3019 0.2703 22.8751

H 10.9823 -0.2190 22.1583

H 12.1905 0.5075 22.7722

H 10.7394 1.0311 22.8935

C 11.3019 0.2703 25.4234

H 10.9823 -0.2190 26.1402

H 12.1905 0.5075 25.5263

H 10.7394 1.0311 25.4050

O 10.8922 3.6489 24.1493

C 11.1274 4.8317 24.1493

C 11.3019 5.6127 22.8751

H 10.9823 5.1234 22.1583

H 12.1905 5.8499 22.7722

H 10.7394 6.3735 22.8935

C 11.3019 5.6127 25.4234

H 10.9823 5.1234 26.1402

H 12.1905 5.8499 25.5263

H 10.7394 6.3735 25.4050

O 17.2847 -1.6935 24.1493

C 17.5199 -0.5107 24.1493

C 17.6944 0.2703 22.8751

H 17.3748 -0.2190 22.1583

H 18.5830 0.5075 22.7722

H 17.1319 1.0311 22.8935

C 17.6944 0.2703 25.4234

H 17.3748 -0.2190 26.1402

H 18.5830 0.5075 25.5263

H 17.1319 1.0311 25.4050

Dipole moments (D)

Methanol

		m052x	m062x	wb97xd
MP Trunc	1	2,2068	2,148	2,0966
	2	2,1852	2,1295	2,0844
	3	1,9923	1,9518	1,9414
	4	2,1141	2,046	2,0169
	5	2,1666	2,1015	2,0779
	6	2,0413	1,9925	1,9967
	7	2,0137	1,9595	1,8949
	8	2,1625	2,0973	2,0731
	9	2,1568	2,0967	2,0766
	10	2,0981	2,0429	1,9792
	11	2,1856	2,13	2,0848
	12	2,1264	2,0771	2,0356
MP	1	2,5985	2,5521	2,5428
	2	2,5501	2,5098	2,4982
	3	2,048	2,0142	1,977
	4	2,49	2,436	2,4357
	5	2,5243	2,4733	2,4722

	6	2,024	1,9747	1,9459
	7	2,3428	2,295	2,2928
	8	2,5237	2,4731	2,4715
	9	2,4724	2,4235	2,4201
	10	2,4531	2,4038	2,4011
	11	2,5531	2,5132	2,5016
	12	2,4476	2,4139	2,3992

Ethanol

		m052x	m062x	wb97xd
MP Trunc	1	2,0601	2,0063	1,9689
	2	2,2157	2,1551	2,1433
	3	2,1886	2,1276	2,1148
	4	2,0306	1,9736	1,984
	5	2,0005	1,9477	1,8995
	6	1,8264	1,7726	1,7591
	7	2,0689	2,0143	1,9502
	8	1,9597	1,9068	1,8591
MP	1	2,3274	2,2953	2,2792
	2	2,4941	2,4572	2,4463

	3	2,4717	2,4349	2,4238
	4	2,0611	2,0076	1,9857
	5	2,1906	2,1502	2,1514
	6	2,0099	1,9669	1,9751
	7	2,2494	2,2057	2,2014
	8	2,148	2,1073	2,1084

Acetonitrile

		m052x	m062x	wb97xd
MP Trunc	1	4,80	4,70	4,72
	2	4,76	4,64	4,69
	3	4,51	4,40	4,44
	4	4,24	4,14	4,16
	5	4,76	4,64	4,69
	6	4,80	4,70	4,72
	7	4,24	4,14	4,16
	8	4,51	4,40	4,44
MP Trunc	1	4,96	4,87	4,90
	2	4,85	4,75	4,77
	3	4,58	4,48	4,51

	4	4,29	4,19	4,22
	5	4,85	4,75	4,77
	6	4,96	4,87	4,90
	7	4,29	4,19	4,22
	8	4,58	4,48	4,51

Pyridine

		m052x	m062x	wb97
MP Trunc	1	2,54	2,45	2,49
	2	2,69	2,60	2,63
	3	2,49	2,39	2,44
	4	2,60	2,54	2,54
	5	2,63	2,53	2,58
	6	2,37	2,30	2,33
MP	1	2,54	2,43	2,48
	2	2,85	2,78	2,80
	3	2,54	2,45	2,50
	4	**2,77**	2,73	**2,74**
	5	2,61	2,49	2,55

6	2,37	2,29	2,33

Acetone

		m052x	m062x	wb97xd
MP Trunc	1	3,0295	3,129	3,114
	2	3,1787	3,2046	3,1846
	3	3,2205	3,3067	3,2887
	4	3,3098	3,2605	3,2375
MP Trunc	1	3,0442	3,3051	3,2981
	2	3,2174	3,2513	3,2646
	3	3,2779	3,481	3,4665
	4	3,3741	3,3581	3,3577
